# A Clinical Trial to Introduce Voluntary Medical Male Circumcision for HIV Prevention in Areas of High Prevalence in the Dominican Republic

**DOI:** 10.1371/journal.pone.0137376

**Published:** 2015-09-14

**Authors:** Maximo O. Brito, Leonel Lerebours, Claudio Volquez, Emmanuel Basora, Shaveta Khosla, Flavia Lantigua, Roberto Flete, Riqui Rosario, Luis A. Rodriguez, Mathius Fernandez, Yeycy Donastorg, Robert C. Bailey

**Affiliations:** 1 University of Illinois at Chicago, Chicago, Illinois, United States of America; 2 Clinica de Familia, La Romana, Dominican Republic; 3 HIV Vaccine Trials Unit, Instituto Dermatologico y Cirugia De Piel, Santo Domingo, Dominican Republic; University of Ottawa, CANADA

## Abstract

**Background:**

Voluntary Medical Male Circumcision (VMMC) is an effective strategy to reduce the risk of HIV infection. Studies conducted in the Dominican Republic (DR) suggest that acceptability of VMMC among men may be as high as 67%. The goal of this clinical trial was to assess the acceptability, uptake and safety for VMMC services in two areas of high HIV prevalence in the country.

**Methods:**

This was a single-arm, non-randomized, pragmatic clinical trial. Study personnel received background information about the risks and benefits of VMMC and practical training on the surgical technique. A native speaking research assistant administered a questionnaire of demographics, sexual practices and knowledge about VMMC. One week after the surgery, participants returned for wound inspection and to answer questions about their post-surgical experience.

**Results:**

539 men consented for the study. Fifty seven were excluded from participation for medical or anatomical reasons and 28 decided not to have the procedure after providing consent. A total of 454 men were circumcised using the Forceps Guided Method Under Local Anesthesia. The rate of adverse events (AE) was 4.4% (20% moderate, 80% mild). There were no serious AEs and all complications resolved promptly with treatment. Eighty eight percent of clients reported being “very satisfied” and 12% were “somewhat satisfied” with the outcome at the one-week postoperative visit.

**Conclusions:**

Recruitment and uptake were satisfactory. Client satisfaction with VMMC was high and the rate of AEs was low. Roll out of VMMC in targeted areas of the DR is feasible and should be considered.

**Trial Registration:**

ClinicalTrials.gov NCT02337179

## Introduction

The World Health Organization (WHO) has recommended that Voluntary Medical Male Circumcision (VMMC) be offered to men as part of a comprehensive package of HIV reduction strategies in areas of high HIV prevalence [1]. This recommendation follows the publication of landmark trials confirming that VMMC is effective in reducing the risk of HIV acquisition [2–4]. Programs to scale- up VMMC are underway in some African countries [5], but limited data exist on the feasibility of introducing this strategy outside that continent.

The Caribbean is the area of the world with the second highest HIV prevalence [6]. In the Dominican Republic (DR), HIV prevalence is not uniform and varies within and between geographic areas. The eastern region of the country, for instance, is reported to have a prevalence of 1.2% [7], but the proportion is significantly higher (3.2%) in a distinct group of communities surrounding sugar cane plantations with large numbers of migrant and temporary workers [8]. A preliminary study conducted in the largest province of the eastern region suggested that knowledge of the benefits of VMMC for HIV prevention was low in men [9]. Only 21% of men knew the benefits of VMMC to decrease the risk of HIV. Hygiene was consistently identified as the most significant benefit of VMMC among those surveyed. The initial acceptability of VMMC was only 29%, but increased to 67% after men received education about the benefits of the procedure.

Using these preliminary data, we designed a pilot study to introduce VMMC in two clinics that serve men at risk for HIV and sexually transmitted infections (STI). The objectives of this study were: to build the capacity required to offer VMMC and to assess the uptake, safety and patient satisfaction with the procedure. To our knowledge, this pilot clinical trial is the first time VMMC has been offered for HIV prevention to adult men outside of Africa.

## Materials and Methods

### Study setting and design

The operative phase of this single-arm, non-randomized, pragmatic trial was conducted from February 2013 to March 2014 at the STI clinic at Instituto Dermatológico y Cirugía de Piel in Santo Domingo (IDCP) and the Clínica de Familia in La Romana (CFLR), Dominican Republic. These sites were selected based on their high number of male clients at risk for HIV and STI infection and the availability of service providers willing to be trained, adequate infrastructure, HIV voluntary counseling and testing services (VCT), and STI treatment.

### Subject recruitment and enrollment

Our plan was to recruit a convenience sample of 500 men since this is was a single arm trial without a control group. Educational and recruitment materials detailing the benefits/risks of VMMC and the study procedures were created before the start of the study. A Community Advisory Board reviewed all study materials and consent documents, and their feedback was incorporated in the final documents. A brochure with postoperative instructions detailing best practices for wound care was also created.

Study participants were recruited by several means. Fliers were posted at bars, nightclubs and communities frequented by young men. Outreach workers distributed educational materials in the community and invited men at nightclubs and bars. Participants were also referred from the STI clinic at IDCP. A de-identified pre-screening form was filled out for every individual who received educational materials or information on VMMC in the field, and sign-in sheets were kept for every educational session. These forms allowed the investigators to estimate the total number of men who received information about the benefits and risks of VMMC.

The study required 3 visits: an initial visit on the day of the consent process, a second visit on the day of the surgical procedure, and a one-week postoperative visit. In some instances, participants were consented and had the surgery on the same day. All subjects participated in group and/or individual educational sessions that reviewed the benefits/risks of VMMC, the surgical technique, potential surgical and post-surgical complications, and went over the study procedures. All subjects were given the opportunity to read the consent form, ask questions, and were given the opportunity to take the consent home and discuss it with their partners or family members, if desired.

For inclusion in the study, men had to be uncircumcised and between 18 and 40 years of age. Exclusion criteria included foreskin covering less than half of the glans, history of bleeding disorders, history of keloid formation and any medical condition or anatomical abnormality that could increase the risk of complications during elective ambulatory surgery. Participants who had a temporary contraindication for surgery were treated and reevaluated once the condition had resolved. Men with anatomic abnormalities of the penis and/or foreskin were referred to the urologist for further evaluation and treatment.

### Collection of behavioral and biomedical data

After enrollment, a behavioral questionnaire was administered and HIV VCT was performed. The questionnaire included questions on demographics, sexual history and practices, high-risk behaviors, history of STIs, penile trauma, beliefs about VMMC and sexual health/hygiene. HIV testing was done using a rapid, qualitative immunoassay for the detection of HIV 1 and 2 antibodies (Alere Determine HIV 1/2, Alere Medical Co., Chiba, Japan or Retrocheck HIV, Qualpro Diagnostics, Goa, India). Those who tested positive were referred for a confirmatory HIV-ELISA and, if confirmed, for HIV treatment. We informed those who tested positive for HIV that VMMC would not be beneficial to them, but gave them the choice to proceed with the procedure or decline participation.

### Training of providers

The training phase consisted of a one-week classroom course imparted to all study personnel using training modules that reviewed every important aspect of male circumcision. Four physicians received surgical training over a period of 3 weeks on the Forceps Guided Method using the WHO Manual of Male Circumcision Under Local Anesthesia [10]. One additional physician was trained at the Nyanza Reproductive Health Society (NRHS) in Kenya. The training was conducted by experienced providers from the NRHS and supervised by a local urologist. Each physician was required to complete 20 circumcisions before allowed to operate independently.

### Adverse events

Wound healing and adverse events (AE) were assessed one week after circumcision or if a participant came with a complaint during an unscheduled visit. All AEs were recorded by the treating physician and communicated promptly to the principal investigator (PI). A committee formed by the PI, the site co-PI, the physician(s) and nurse(s) who performed the procedure, and the research assistants at the respective site, reviewed all complications and determined if they were true adverse events related to the procedure according to the research protocol. Once the complication was deemed an AE, the committee proceeded to categorize it as mild, moderate or severe. AEs were reported to the IRB and ethics committees during scheduled protocol reviews.

### Amendments/changes to the original study protocol

The authors made the following major changes to the initial study protocol: age range of eligibility was changed from 18–35 years to 18–40 years to reach a larger segment of the population; the criteria for classifying wound infections after circumcision was modified to be more specific and user friendly; and the initial enrollment number approved by the IRB was increased from 525 to 575 subjects.

### Statistical methods

#### Study outcomes

The primary outcome of this study was the uptake/acceptability of VMMC as measured by the proportion of men who agreed to be circumcised after being offered. Secondary outcomes were the number and percentage of adverse events by severity (mild, moderate, severe) and the proportion of clients satisfied with the procedure.

#### Data analysis

Measures of central tendency and dispersion were calculated for numeric variables and frequencies were obtained for all variables. Some variables were dichotomized based on the presence (yes) or absence (no) of the characteristic evaluated. Study data were collected and managed using REDCap electronic data capture tools hosted at the University of Illinois at Chicago. REDCap is a secure, web-based application designed to support data capture for research studies, providing an intuitive interface for validated data entry and automated exports procedures of data downloads to SAS. Data was analyzed using SAS 9.1.3 (SAS Institute Inc., Cary, NC, USA)

### Ethics statement and trial registration

All participants were informed of the risks and benefits of the study and provided signed informed consent. Study procedures were performed in accordance to local laws and institutional guidelines for the appropriate treatment of human subjects were followed. The research protocol (S1 Protocol) was reviewed and approved by the Institutional Review Board (IRB) of the University of Illinois at Chicago, the National Council on Bioethics of the Dominican Republic and the Ethics Committee of the Instituto Dermatologico y Cirugia de Piel in Santo Domingo, Dominican Republic. No committee or review board has imposed restrictions on the data.

The authors confirm that all ongoing and related trials for male circumcision have been registered or are already published in the scientific literature. This study was registered late because the authors were unaware that this single-arm, non-randomized, pragmatic trial qualified as a true clinical trial until informed by this journal’s editor. The clinical trial was registered at ClinicalTrials.gov (registry # NCT02337179) (S1 TREND Checklist)

## Results

### Training phase

A total of 96 patients presented for VMCC during the training of providers. The median age of this group was 26 years (IQR 21–35). Twelve men were excluded and 84 were circumcised by trainees with 7 (8.3%) resulting in AEs, all of which were mild and resolved within days. Eighty percent of participants were “very satisfied” and 20 percent were “somewhat satisfied” with the procedure at the post-operative visit. None were dissatisfied.

### Study phase

**Fig 1:**
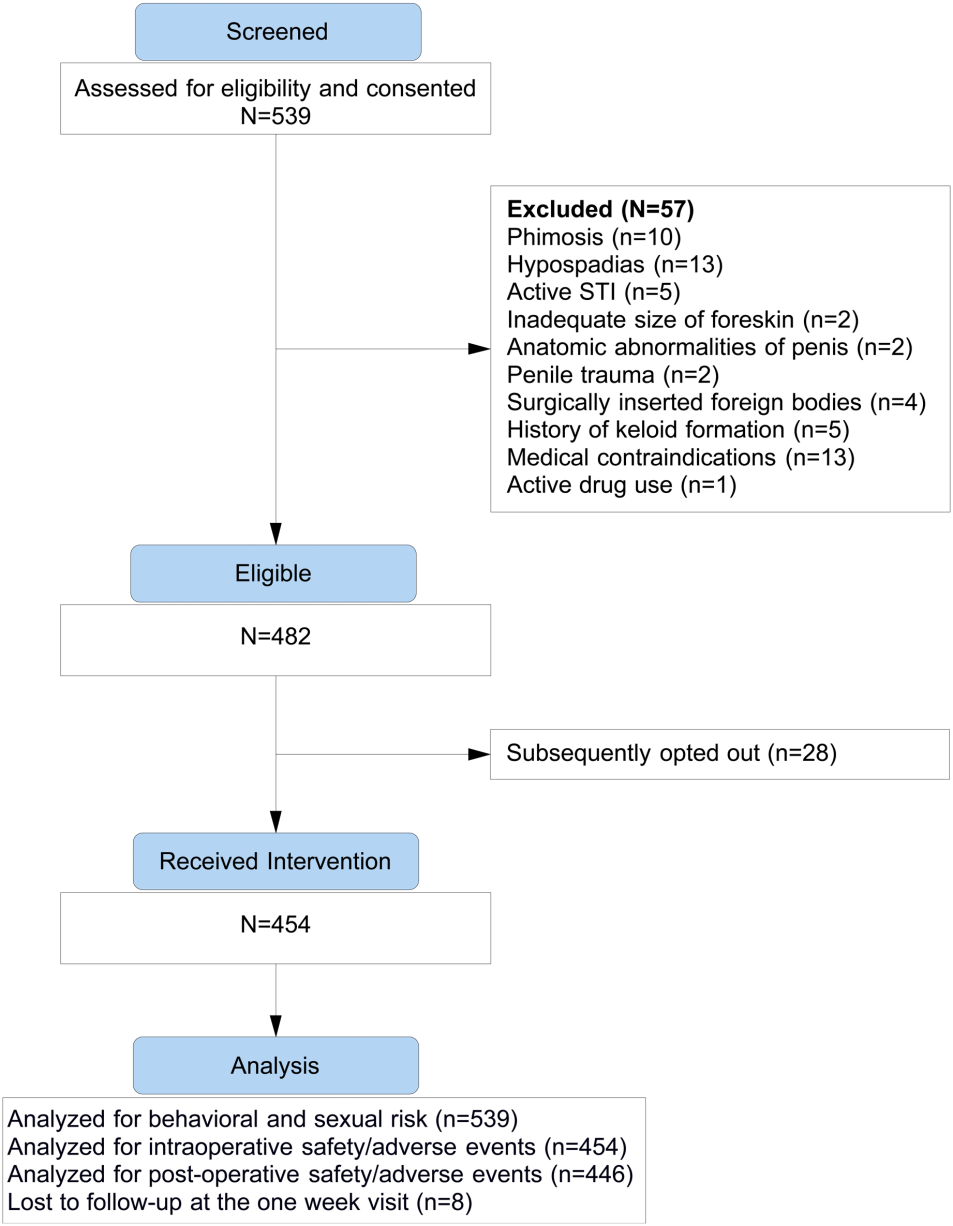
Study Flow Diagram.

Approximately 952 men were approached by our outreach workers and/or attended scheduled educational/informational sessions for this study. Of these, 539 (57%) were assessed for eligibility and were consented for the study (Fig 1). Table 1 describes socio-demographic characteristics of the men who consented to participate.

**Table 1:** Socio-Demographic Characteristics and Behaviors.

Variable	Men recruited and consented N = 539	Men circumcised N = 454	Men consented but not circumcised N = 85	P value**
Study Site, n (%)				
*IDCP* *	228 (42)	200(44)	28 (33)	0.06^a^
*CFLR* ǂ	311 (58)	254 (56)	57 (67)	
National origin, n (%)				
*Dominican*	527(98)	445 (98)	82 (99)	1.00^b^
*Haitian*	9(2)	8 (2)	1 (1)	
Median age (range)	26 (18–40)	26 (18–40)	27 (18–40)	0.60^c^
Median last grade at school (IQR)¥	12^th^ (10–12)	12^th^ (10–12)	12^th^ (9–12)	0.93^d^
Median age at first intercourse (IQR)	16 (14–17)	16 (14–17)	15 (14–16)	0.11 ^d^
Median no. of female partners last 30 days (IQR)	1 (1–2)	1 (1–2)	1 (1–1)	0.97 ^d^
Partnered or married, n (%)	342 (64)	298 (66)	44 (53)	**0.03** ^a^
Never use condoms, n (%)	85(16)	75 (17)	10 (13)	0.39^a^
Sex with men, n (%)	28 (5)	21 (5)	7 (9)	0.14^a^

** P values are for the comparison of men circumcised (454) and men consented but not circumcised (85).

^a^ p value from Pearson’s chi square test

^b^ p value from Fisher’s exact test

^c^ p value from two sample t test for difference of means

^d^ p value from Wilcoxon Rank Sum Test

*Instituto Dermatologico y Cirugia de Piel

^ǂ^ Clinica de Familia, La Romana

^¥^ Interquartile Range

Median age was 26 years (IQR 22–32, range 18–40) and the median age of their first sexual encounter was 16 (IQR 14–17). Ninety eight percent (527/537) of participants were Dominican and 2% were Haitian. Eleven percent (57/539) were excluded from participation due to a medical or anatomical contraindication and 5% (28/539) decided not to have the procedure after providing initial consent (Fig 1). Ninety six percent (520/539) of those consented agreed to be tested for HIV, including 98% (445/454) of participants who were circumcised. Three men tested HIV positive, 2 already knew their serostatus and 1 was newly diagnosed. Two tests were indeterminate and, in both cases, participants refused to have confirmatory serology.

Twenty five percent (136/539) of participants had ≥ 2 sexual partners over the preceding 30 days and 70% (378/539) had ≥ 2 sexual partners over the preceding year. Only 23% (122/534) of men reported consistent condom use. When asked more specifically about their condom use practices with non-regular partners, 64% (341/530) reported always using a condom. Sixteen percent (86/533) of men reported paying or exchanging gifts for sex during the preceding 6 months. One fifth (103/533) reported consuming alcohol on their last sexual encounter. Twenty three percent (120/532) reported having anal sex with their partners. A third (185/531) reported applying topical substances on their penis to enhance their sexual potency and 6% (28/467) reported using illegal drugs in the preceding year. None of the participants had used intravenous drugs. Five percent (28/535) reported having had sex with other men.

With varying degrees of frequency, participants reported having experienced swelling (36%, 191/532), abrasions (60%, 319/532) and bleeding (19%, 99/532) of the penis during intercourse at some point prior to their VMMC. To understand the participant's sexual performance for the 6 months prior to VMMC we asked if certain symptoms had been present for a period longer than 2 weeks. In this category, 17% (88/506) had experienced loss of interest in sex, 36% (181/506) had instances in which they could not reach an orgasm and 55% (278/506) reported premature ejaculation. In addition, 22% (111/504) reported pain during intercourse, 52% (262/506) did not derive pleasure from sex and 34% (170/503) experienced erectile dysfunction.

Participants reported better hygiene (29%), STI risk reduction (26%), HIV risk reduction (20%) and improvement of their sexual experience (19%) as their primary reasons for becoming circumcised. There was some overlap between these categories with 10% of those listing hygiene as their primary reason for agreeing to VMMC also stating that they were doing it for STI/HIV risk reduction.

### Circumcision

A total of 454 men were successfully circumcised over a period of 12 months in this pilot study. The median duration of the procedure for all practitioners was 38 minutes (IQR 30–45). Participants resumed their normal activities at a median of 3 days (IQR 2–4) and 78% had resumed their usual activities by the one-week postoperative visit. There were a total of 20 complications in 17 men for an overall AE rate of 4.4% (Table 2). Four (20%) complications occurred peri-operatively and 16 (80%) occurred within the first month after surgery. Of the 16 postoperative complications, 14 were diagnosed at the one-week postoperative visit and 2 were diagnosed later: one participant bled 12 days after the procedure and another participant sustained a dehiscence while having sex 23 days after the procedure. There were 4 (20%) complications of moderate severity and the remainder were mild. In at least 3 cases (2 dehiscences and 1 excessive swelling) we believe the complication resulted from resumption of sexual activity earlier than recommended. One participant experienced delayed healing due to genital trauma while riding a motorcycle. Four men needed resuturing. All complications resolved promptly without leaving permanent sequealae. There were no significant differences in demographics and sexual risk profile at baseline between the men who experienced AEs and those who did not.

**Table 2:** Adverse Events.

	N = 20	Severity
Dehiscence	5	1 moderate, 4 mild
Bleeding	4	2 moderate, 2 mild
Excessive swelling	2	Mild
Infection	2	Mild
Anesthesia-related events	2	Mild
Hematoma	2	1 moderate, 1 mild
Excessive pain	1	Mild
Delayed healing	2	Mild

A total of 446 participants (98%) returned for the one-week postoperative visit. Eight participants were lost to follow up. At that visit, 88% (390/444) of participants reported being “very satisfied” and 12% (53/444) were “somewhat satisfied” with the outcome of the procedure. One client was “somewhat dissatisfied” and none were “very dissatisfied”. The client who was “somewhat dissatisfied” did not experience any adverse events, but reported "pain and friction of the suture with my underwear" as the main reason for his dissatisfaction.

## Discussion

The results of this pilot study suggest that VMMC can be offered in the outpatient setting, with low rates of AEs, to adult Dominican men as part of a comprehensive package of HIV reduction strategies. Approximately 57% of individuals who received information about VMMC or who attended one of our educational sessions consented to participate in the study which entailed agreeing to circumcision. This percentage is close to the 67% intention to become circumcised obtained in our preliminary acceptability study. Similarly, hygiene, the most cited primary reason for having a VMMC in this study, was significantly associated with the willingness to be circumcised in our prior surveys [9, 11]. Other studies have shown high acceptability of the procedure in Caribbean [12], Chinese [13], and Indian men [14], but to our knowledge, this is the first attempt to offer VMMC services outside of Africa. Although our previous acceptability study found that Haitian men would be more willing than Dominicans to become circumcised, we did not recruit a significant number of Haitian nationals. This was due to the lack of recruitment and educational materials in Creole which limited enrolment to Spanish speakers. Larger scale and better funded programs will need to include materials translated to Creole and a more targeted strategy to increase uptake among Haitian men.

We recruited a large number of participants with at least a higher than average risk of acquiring HIV. In the most recent Demographic and Health Survey (DHS) of the DR [7], a quarter of men between the ages of 15 and 39 years reported having ≥ 2 sex partners over the preceding year. By contrast, 70% of participants in our study reported the same number of partners over a similar time period. In addition, 16% percent of men in our study reported paying for sex or exchanging gifts for sex over the preceding 6 months compared to 4.2% of men surveyed in the DHS. The percentage of men with ≥ 2 sexual partners who reported using a condom during their last sexual encounter was similar in the DHS (49%) and in this study (47%).

A surprising number of men reported having experienced problems with the penis and foreskin at some point prior to VMMC. One fifth had experienced bIeeding during intercourse and more than 30% reported abrasions and swelling of the foreskin. Twenty two percent reported pain during intercourse and 34% had erectile dysfunction. Coital injuries have been reported to occur more frequently in uncircumcised [15] and these men may be the early adopters of VMMC. Similar findings were observed in another study where men who elected to be circumcised were more likely to report preputial problems and anatomical abnormalities such as deviation of the penis during erection, difficulty inserting the penis during intercourse and difficulty achieving an erection due to skin tightness [16].

The rate of AEs was 4.4% in this trial. The majority (80%) of these complications were mild and resolved promptly. Four cases required re-suturing, but healed satisfactorily without further complications or permanent sequelae. Our AEs fall within the range observed in the African randomized clinical trials where rates of complications were 1.7% [3], 3.8% [2], and 8% [4]. Although the proportion of complications was higher in this trial than in the Kenyan trial [3], the AE rate reported for that trial's first 479 surgeries was 3.5% [17], which is closer to our number and a more appropriate comparison for the level of experience of our clinicians. Our rate of AEs decreased from 8.3% during training to 4.4% in the actual study. This progressive decrease in complications with increasing expertise of the providers is similar to reports from Rakai, Uganda where the rate of moderate-severe AEs decreased from 8.8% for the first 20 unsupervised procedures to 4% for the next 20–99 procedures [18]. In that study, providers averaged 40 minutes to complete the first 80–100 procedures and 27.1 minutes for the second 100 procedures. In the Kenyan study, the median time to complete a circumcision decreased from 38 minutes for the first 100 procedures to 21 minutes after 1400 procedures [19]. The median surgical time in our trial was 38 minutes (IQR 30–45) for the entire study and did not decrease significantly by quartiles. This was probably due to the way we staggered physicians to start operating at different times during the study. In addition, one of the sites began circumcising 2 months before the other, and thus, when physicians at that site were becoming proficient and taking less time to complete a circumcision, the slow pace of the ones starting later increased the average surgical time for the entire group. There was no difference in surgical times between study sites (p = 0.6).

The main strength of this study is that it provided proof of concept that VMMC can be safely offered in the DR. It also proves that Dominican men are willing to be circumcised if the benefits and risks are clearly presented. We were able to recruit a relatively large sample size and observed low rates of complications. By doing this study, we contributed to build capacity and infrastructure for future VMMC programs. This study has several limitations. It is plausible that men recruited at these two urban clinics are not representative of all Dominican men. The large number of participants with baseline coital injuries and anatomic problems of the penis may indicate a bias towards men who knew they had a problem and wanted to correct it. We also recruited a very low number of Haitian participants. In addition, the study lacks a full cost-effectiveness analysis of the intervention and we don’t have long term follow-up client satisfaction and adverse event data. We should note that in large clinical trials most adverse events occurred within a few weeks after circumcision [3]. All participants with adverse events were evaluated at our study sites regardless of when the complications occurred. Although risk compensation, the potential increase in higher risk behaviors due to a perceived risk reduction, has been mentioned as a theoretical long term adverse effect of VMMC, no studies to date have found risk compensation after VMMC [20]. The absence of an independent Data and Safety Monitoring Board to evaluate and grade adverse events could also be considered a limitation.

Offering VMMC services was a unique opportunity to bring young Dominican men, a hard to reach population that is typically alienated from the healthcare system, to primary care clinics. We believe that all services provided to participants in this study (97% of men receiving VCT for HIV, counseling on STI reduction, medical exams and free condoms) contributed to improve their sexual health. Thus, per WHO/UNAIDS guidelines, we should seize the opportunity to continue to use these services as a way to engage and retain men in care in target areas of moderate to high HIV prevalence where large numbers of uncircumcised men reside. The Ministry of Health should explore offering VMMC on a larger scale to Dominican men.

## Supporting Information

S1 Protocol (PDF)

S1 TREND Checklist (PDF)
