# Supplementary material for: A Clinical Trial to Introduce Voluntary Medical Male Circumcision for HIV Prevention in Areas of High Prevalence in the Dominican Republic
Source: PLoS One. 2015 Sep 14;10(9):e0137376. doi: 10.1371/journal.pone.0137376 (PMC4569265; doi:10.1371/journal.pone.0137376)
Supplement: S1 Protocol — (PDF) [file pone.0137376.s001.pdf]

S1. Study Protocol

A pilot study to introduce male circumcision (MC) services to prevent HIV infection in two high prevalence areas of the Dominican Republic (DR)

Maximo Brito, Yeycy Donastorg, Claudio Volquez, Leonel Lerebours, and Robert C. Bailey.

Abstract

Voluntary Medical Male circumcision (VMMC) is an effective strategy to reduce the risk of HIV acquisition in heterosexual men. Observational data and randomized controlled trails (RCTs) conducted in Africa[1,2,3] suggest that the procedure reduces the risk of HIV acquisition by 50-76% in heterosexual males. VMMC has also been shown to reduce the incidence of other sexually transmitted infections (STI), including herpes simplex virus type 2 (HSV-2) and human papilloma virus (HPV) in men[4], and Chlamydia[5], trichomonas vaginalis (TV), bacterial vaginosis (BV) and HPV in their female partners [6].

The results of the RCT's are generating increased demand for safe and affordable MC services in areas of moderate-to-high HIV prevalence where the MC rates are low. Latin America is a region where circumcision of males is uncommon[7]. In the Dominican Republic (DR), which is the focus of this study, a 2007 nationwide household survey found that only 13.7% of men between the ages of 15-59 were circumcised[8].

AIDS is the leading cause of death in the Caribbean basin among adults aged 15-44 years. There are approximately 240,000 people currently living with the disease, most of them residing in the Dominican Republic (DR) and Haiti[9]. The prevalence of HIV infection in the DR is approximately 0.8% nationwide[8] but it is higher in selected high-risk populations, such as among people living in the "bateyes", the small towns surrounding sugar cane plantations, where the prevalence is 3.2%[10]. In pregnant women seeking prenatal care at the main hospital in the Altagracia province HIV seroprevalence is 4.5 %[11]. One of the areas of highest prevalence (1.2%) in the country is Region V of health, which includes the provinces of La Altagracia, El Seibo, Hato Mayor, La Romana and San Pedro de Macorís. Prevalence is also higher in the urban slums of the capital city, Santo Domingo.

The public health impact of introducing MC as a strategy to prevent HIV/STI transmission in areas of high HIV/STI prevalence and low MC rates can be significant. In addition to decreasing the rates of HIV and other STI, the intervention could indirectly help decrease the rates of HPV associated cervical cancer. The study proposed here is to develop, implement, monitor and evaluate pilot MC services in selected sites to assess the acceptability, uptake, safety and demand for these services, and to use these data to develop, in collaboration with the Dominican Ministry of Health (MoH), a proposal to the National Institute of Mental Health (NIMH) for support of operations research concerning the safety, uptake, and efficacy of MC interventions in the DR.

a) Specific Aims:

Based on the results of numerous observational studies and the three RCTs the World Health Organization (WHO) and the Joint United Nations Programme on HIV/AIDS (UNAIDS) have endorsed VMMC as a biomedical intervention for HIV prevention. As such, countries with a high prevalence of HIV in the population are encouraged to examine the feasibility of offering MC services as part of the portfolio of HIV prevention strategies at a national level. The specific aims for this project are:

1) To develop culturally appropriate education materials about the benefits of VMMC for clients attending pilot healthcare centers.

2) To train a core group of providers on proper surgical and counseling techniques to provide comprehensive VMMC services.

3) To determine the acceptability, uptake and demand for MC services in key areas of high HIV prevalence.

H1: The acceptability and uptake among high risk men will be around 60%.

4) To assess the safety and adverse events of performing circumcision in resource constrained settings.

H2: VMMC will result in a complication rate of less than 2%.

5) To assess sexual risk behaviors, perceptions of sexual function and sexual pleasure in men before and after VMMC. H3: there will be no differences in risk behaviors, sexual function and pleasure after VMMC

6) Estimate point prevalence of most common STI in a subset of circumcised men and preserve a small sample of blood to test future scientific hypothesis and/or newer testing techniques as they become available.

7) To build collaborations with the MoH and other partners in the DR and at UIC to develop a proposal to NIMH for operational research in the context of larger scale MC service provision.

b) Background and significance:

b.1. Male Circumcision to Prevent HIV Transmission

Male circumcision is perhaps one of the oldest surgical procedures performed on humans. The millenary practice of removing the male foreskin has cultural, religious and medical dimensions that have been widely studied. Around the world, the procedure is

mostly performed for religious reasons in persons of the Jewish and Muslim faiths. Medical indications for MC include phimosis, paraphimosis and balanitis, among others[12]. Early evidence of a relationship between MC and HIV infection came from a number of smaller, mostly observational studies, done in Africa[13]. These historical data were validated with the completion of three large RCTs in South Africa, Kenya and Uganda, which have shown an overall decrease of 50-76% in the risk of acquiring HIV infection in circumcised males when compared to controls[1,2,3].

The pathogenesis of the initial infection at the level of the foreskin involves tissue macrophages, cell receptors and a moist, poorly keratinized inner prepuce.
The first cellular target of the HIV virion on the human genitalia are the tissue dendritic cells in the lamina propria of the submucosa[14]. These cells express surface CD4 receptors and CCR5 co-receptors for which HIV has high affinity. In intact human skin, the dendritic cells are shielded from the outer environment by the relatively thick layer of keratin covering the surface of the epithelia. Tissue studies of the human foreskin have shown that the inner lining of the prepuce, exposed during a penile erection, has a thin keratin layer that is very susceptible to microscopic abrasions and can expose the tissue macrophages making them vulnerable to infection by HIV[15].

b.2. MC for STI prevention:

MC is an effective strategy to decrease the risk of infection with HIV and other STI in heterosexual males. A beneficial effect has also been observed in decreasing the risk of STI in females. The RCTs confirmed that VMMC decreases herpes simplex virus type 2 (HSV-2) acquisition by 29%, and HPV prevalence by 34% in men[4]. Among female partners of circumcised men, bacterial vaginosis was reduced by 40%, and trichomonas vaginalis infection was reduced by 48%[6]. Genital ulcer disease was also reduced among males and their female partners by approximately half[16].

b.3. HIV in the Caribbean and the Dominican Republic

The significant decrease in the rate of HIV acquisition observed among circumcised males who participated in the African RCT is generating an increased demand for safe and affordable VMMC services in areas of high HIV prevalence where the procedure is not routinely performed. Latin America is one of such areas where routine circumcision of males is uncommon[7]. In the Dominican Republic, only 13.7% of men between the ages of 15-59 years are circumcised[8]. The Caribbean is the region of the world with the highest HIV prevalence outside of sub-Saharan Africa (1.0%)[9]. Approximately 240,000 individuals are currently living with the disease. Among the Caribbean countries, the Dominican Republic and Haiti report the highest prevalence in the region. According to data from a recent In the DR, the prevalence of HIV infection is 0.8% nationwide[8], however, the numbers are higher in selected populations and in certain geographic locations.

The Dominican healthcare system is administratively divided in 9 regions, which include the country’s 31 provinces. Region 0 of health includes the National District and the

provinces of Santo Domingo and Monte Plata. The city of Santo Domingo is the capital and financial center of the DR. It has an estimated population of 2.7 million people spread in an area of about 1,400 KM^2. 90% of the population lives in an urban setting in this province[17]. The overall prevalence of HIV is around 0.7 % in the entire region but is higher in vulnerable groups such as commercial sex workers (CSW) (3.3%), Men who Have Sex with Men (MSM) (5.9%) and drug users (7.1%)[18].

Regions V and VII report the highest prevalence of HIV in the country, 1.2 % and 1.5% respectively[8]. Specific populations within these provinces have rates of HIV considerably higher than the national average. For instance, prevalence is 3.2% in the bateyes, the small communities surrounding sugar cane plantations, where a mixed population of Dominicans and Haitian migrant workers cohabitate. Our team has conducted preliminary studies in the Altagracia Province, part of Region V, since it reports the highest province-specific HIV rates and the lowest circumcision rates in the country. A survey of pregnant women seeking prenatal care at the main hospital in the province found an HIV seroprevalence of 4.5 %[11]. The prevalence of HIV is 5.2% in CSW, 7.6% in MSM and 8.5 % among drug users[18].

c) Preliminary studies:

The parent study for this project is a RCT of VMMC to reduce HIV incidence in Kisumu, Kenya whose principal investigator is Prof. Robert C. Bailey, a co-investigator in the current proposal[3]. The main objective of that trial was to assess the effectiveness of VMMC in reducing HIV incidence in young, sexually active men. The protective effect found in an intent-to-treat analysis was 53% and in an as treated analysis adjusting for cross-overs and men found to be HIV seropositive at baseline estimated the protective effect to be 60%. Adverse events related to the surgical procedure were few, just 1.5%. Behavioral disinhibition (risk compensation) on the part of circumcised men was small and not significant.

Drs. Brito and Bailey conducted a feasibility study of healthcare facilities in the Altagracia province in the Dominican Republic during June and July of 2007. A total of 37 healthcare facilities were surveyed. The study found that most facilities lacked appropriate equipment to perform surgical procedures and only four (11%) had a surgical theater. 43 healthcare workers, the majority of them physicians (91%), were interviewed for the study. Only 23 % of the personnel had experience performing MC and a significant number of providers (76%) believed they needed comprehensive training in the procedure prior to starting a MC program. Quantitative and qualitative studies to assess the acceptability of MC among men, women and providers in the Altagracia Province were conducted in 2008. In these studies, 73% of men thought that MC improved hygiene and approximately one third knew that it reduces the risk of penile cancer or STI. Only a small percentage (21%) knew that MC helps prevent HIV infection. When first asked, 29% of participants reported that they would be willing to be circumcised if the procedure was offered to them. However, after an educational session detailing the benefits of the procedure for HIV and STI prevention, the acceptability increased to 67%. Such increment was also observed in previous studies

done in Botswana[19] and Thailand[20] where acceptability increased from 61 % to 81% and from 14% to 66%, respectively, after a similar information session. In multivariate analysis, the strongest predictors of men's acceptability of circumcision, before the information session, were Haitian nationality (OR=1.86, 95% CI 1.01-3.41), thinking that circumcision improves hygiene (OR=2.78, 95% CI 1.29-6.0) and not believing that having a circumcision decreases sexual pleasure (OR=2.18, 95% CI 1.20-3.94). These results have been published in the Public Library of Science-ONE[21] and presented at the International AIDS meetings in 2007 and 2009 in Mexico[22] and South Africa.

We also conducted 13 focus group discussions (FGD), 6 with women and 7 with men, each consisting of 6-10 participants (mean=7.9, SD=1.3). One additional FGD was conducted with six physicians, two males and four females, working in rural clinics around the province. Findings of this qualitative study showed that a significant number of participants might be willing to be circumcised, or agree with circumcision for their partners, to improve hygiene, prevent diseases and treat medical conditions, such as phimosis, which can result in pain during intercourse. These results are consistent with our quantitative study on men's acceptability of MC. Perhaps one of the most important findings of the FGD was the enthusiastic endorsement of MC by women, a fact that has potential implications for the successful implementation of a circumcision program in the country (manuscript accepted for publication to AIDS Care.)

Overall, our preliminary data suggest that men are willing to be circumcised provided they receive information about the benefits of the procedure. Before recommending the introduction of VMMC services as part of a comprehensive package of HIV and other STI prevention strategies, these findings need to be confirmed by studying the uptake of VMMC among sexually active men in selected clinics that serve clients in high prevalence areas. In addition, there is need to identify potential barriers to offering MC services in high risk communities prior to proposing to implement a large-scale intervention. In order to properly address these concerns, we will conduct a pilot study that will provide the necessary preliminary data to design a proposal to the NIMH to conduct operational research around a large-scale implementation project.

d) Experimental design and methods

d.1. Pilot study design

The study will be conducted at two sites: the STI clinic at Instituto Dermatologico and Cirugia de Piel (IDCP) in Santo Domingo and the Clínica de Familia in La Romana, Dominican Republic. These sites have been selected on the basis of: 1) high numbers of male clients at risk for HIV and STI infection in communities served by the clinics; 2) high level of acceptability of MC among men in the communities served by the clinics (bateyes of Altagracia); 3) the availability of service providers willing to be trained; 4) the availability of equipment; and 5) the availability of infrastructure, including a minor surgical theater, sterilization facilities and HIV voluntary counseling and testing (VCT) and STI management.

Specific aim #1:

During the first phase of the study, culturally appropriate educational material will be created in Spanish by the principal investigator and local collaborators. Focus Group Discussions (FGD) and in-depth interviews will be conducted with members of the community to learn the educational needs of the community and to assess their opinions on how to best deliver the information on MC that will be contained in the educational materials.

FGD and in-depth interviews

Recruitment of participants

We plan to conduct the FGD in the communities served by the Instituto Dermatologico and the Clinica de Familia, the two main study sites. The plan is to assemble groups of 6-12 participants from the population living in these communities. Community leaders, health promoters , teachers and primary care physicians will be asked to help identify and invite volunteers from their respective communities to participate in the study. There will be no difference in the recruitment process for men and women. They will be recruited from the same venues (schools, clubs, community centers) . Participants will be invited to participate by research personnel with the help of local leaders. These local leaders may be of both genders and will help recruit participants from either gender. The focus groups will be gender-specific. The moderator will explain the purpose of the study to participants. Verbal consent will be obtained from all participants and an information sheet outlining the objectives of the study, their privacy and confidentiality rights and our contact information will be distributed. The document will highlight the voluntary nature of the subject's participation and their right to leave the discussion at anytime if they so desire. The study meets the definition of "minimal risk", therefore, we are requesting a waiver of written consent. We believe this will further preserve the confidentiality of the participants.

The goal is to conduct no more than 15 FGD. Participants in the FGD will be divided by gender. We plan to recruit approximately the same number of men an women for the FGD .

In addition to the FGD , several in-depth individual interviews may be conducted using the same guide than in the FGD.

Eligibility criteria for FGD and interviews

Inclusion criteria:

1) Men and women between the ages of 18 and 40.

Exclusion criteria:

1) Individuals with speech or hearing impairment.
2) Children less than 18 years of age.

Collecting, Processing and Analyzing the data obtained from FGD and interviews

A script will be followed to conduct the FGD. The discussions will be stimulated by using a list of carefully crafted questions on the subject of MC. These questions are designed to obtain information on the following aspects relevant to our investigation:

1) Perception of the population about the frequency of MC in community men.
2) Opinions on the best way to educate men in the community about the benefits of circumcision
3) Opinions about the content of the educational materials educational and how information should be delivered
4) Where should these educational material be posted or distributed to capture the most number of men
5) Opinions on the best venue to organize educational sessions featuring a circumcised man that would be available to answer question from men in the community about his satisfaction with his circumcision
6) Perceptions about who would be a good person to educate men about the benefits of circumcision in the community
7) Opinions on the best way to involve women in the community in a campaign to recruit men for circumcision

The moderator and a research assistant (RA) will lead the discussions. All FGD will be audiotaped and detailed notes will be taken. The content of the recordings will be transcribed shortly after the end of the meeting. The PI ,a native Spanish speaker, will be responsible for translating the recordings from Spanish to English. The English-version transcripts will be coded using the program ATLAS.ti vers. 5.2 based on template of topical categories drawn from questions and issues covered in the discussion guides and from themes emerging from the discussions themselves.

The interviews will be conducted by one of the co-investigators. Verbal consent will be obtained and a document containing information about the study will be provided to the participants.

All data obtained during this study, including recordings and notes will be de-indentified. The names of the participants will not be recorded to protect their confidentiality.

Data Management

All notes, audio recordings and study material obtained during the FGD and interviews will be kept in a locked filing cabinet in an office at the Instituto Dermatologico, Dominican Republic and in the PI's office at the UIC campus upon the investigator's return to Chicago. All data will be de-identified to preserve confidentiality. Names will not be recorded. Verbal informed consent will be sought; there will be no signed consent forms to identify individuals. The participants have the right to review the audio recordings. If a participant requests to listen to the recordings, one of the co-investigators will schedule an appointment for the subject to come to the research site and, in an unoccupied office, will playback the recording for him/her and answer all the questions that the subject may have. If the participant wishes to erase part or all of his/her responses, it will be done on the spot, in his/her presence, using the digital recorder.

The data obtained during these FGD and interviews will inform the design of the educational materials. Once created, these materials will be tested for clarity and appropriateness of language by soliciting input from community health promoters and peer advocates working at the IDCP. Once it is determined that the educational materials are suitable to distribute to clients, we will submit to the Institutional Review Board for approval. Once approved, pamphlets and posters will be printed and distributed at the site clinics and the surrounding communities

<u>Specific aim #2:</u>

During the second phase, selected providers will be trained. This will include counselors in HIV counseling and testing, and medical doctors by an experienced urologist on the appropriate technique for VMMC using the "Manual for Male Circumcision Under Local Anesthesia" published by WHO, UNAIDS. Supervision and evaluation of trainees will be conducted following the protocol used during the parent RCT.

<u>Specific aim #3</u>

Subject recruitment

Study subjects will be recruited in a variety of ways. In Santo Domingo, fliers will be posted in bars and night clubs frequented by men at high risk for HIV/STI acquisition. A group of volunteer commercial sex workers working on an HIV vaccine trial will be asked to refer their clients for the study. Men seeking care for STI at the clinic will also be invited to participate. In La Romana, men attending the Clínica de Familia will be invited to participate. In addition, a group of outreach workers will visit the "bateyes", the communities surrounding sugar cane fields where migrant laborers live, to educate men about MC and invite them to participate in the study. Subjects will be compensated for transport.

The inclusion criteria for the study will be:

1) Uncircumcised men;
2) Aged 18-40 years;

Exclusion criteria include:

1) Foreskin covering less than half of the glans;
2) Bleeding disorders;
3) Keloid formation;
4) Other conditions that might unduly increase the risks of elective surgery.

Men between 18-40 years will be provided with information about the study and will be invited to participate. If they express an interest in participating, informed consent will be obtained. The participant will go over the consent form with the counselor, who will

assess his level of understanding and answer any questions. If there are no temporary or permanent excluding conditions, the participant will be asked to give informed consent for enrollment in the study (appendix A). Men who elect to be circumcised will be fully informed about the benefits and risks of the procedure, as well as the surgical method to be used. After the informed consent is signed, a "Locator Information Form" (form 1) containing confidential data such as subject's name and address will be completed. Only selected research staff will have access to this information, which will be filed separate from the rest of the study forms. A de-identified "demographic information form and behavioral questionnaire" (form 2) will be completed next.

Testing for HIV infection will be carried out using a rapid test. The test kits to be used are the synthetic peptide test, Determine HIV 1/2, and the recombinant antigen test Unigold HIV 1/2 Recombinant. Results are provided to the clients, with appropriate counseling, at the same visit. Participants are taught to observe, read and interpret the tests for themselves in the company of the counselor. Men with positive results will be informed of their HIV status and will be followed-up at the study clinic and referred for a confirmatory HIV-ELISA and, if confirmed, for HIV treatment.

10 ml of blood and a urine sample will be collected at the one-week post circumcision visit. Blood will be centrifuged and two aliquots of 2 ml each will be obtained. One aliquot will be used for all study tests and the other will be preserved for future testing as outlined below and on section d.3. Serologic testing for HSV-2, hepatitis B surface antigen (HBV), and hepatitis C antibody (HCV) will be performed using an ELISA test. A serum Rapid Plasma Reagin test (RPR) will be performed to test for syphilis. Chlamydia and gonorrhea testing will be performed in urine using a Polymerase Chain Reaction (PCR) assay.

All participants who test positive for Chlamydia, syphilis and gonorrhea will be offered treatment free of charge. Participants who test positive for HSV-2 and have active genital lesions will be offered treatment free of charge. Those who test positive but have no active lesions will be counseled on safe sex practices and the risk of transmission to their partners as there is no recommended treatment for asymptomatic individuals infected with HSV-2. HIV, HBV, and HCV positive participants will be referred for evaluation and treatment at Clinica de la Familia in La Romana as these are chronic diseases that require long term treatment usually covered under government programs. If a participant is found to have a treatable STD and he does not return to the site, he will be called by the study investigators using the phone number provided by the participant at enrolment to ensure treatment is provided.

An aliquot of the patient's serum (2 mls) will be preserved for future testing at the University of Illinois. As long as the investigators hold personal identifiable information, participants will be informed of the results of any future testing performed on their blood and will be offered treatment free of charge and referrals as outlined above. Section d.3 provides a complete description of how these samples will be managed, preserved and transported.Urine will not be preserved for future testing.

If there is a temporary, medically curable condition present, such that it would be advisable to defer potential surgery, then the participant will be treated and asked to return for re-evaluation for study eligibility when he is cured. Men found to have an STD or to be HIV-positive will be referred for immediate care. Once enrollment is completed, participants will be given an appointment for the surgery.

Circumcision will be performed soon after informed consent, preferably within a few days. All surgeries will be done under local anesthesia in a clinic by trained clinicians, using the standardized forceps-guided method described by Krieger and colleagues and contained in the WHO MC manual[23]. Circumcised men will be counseled to refrain from sex for six weeks post-surgery and counseled about risk of HIV infection through open wounds during the healing process. They will be checked for complications and asked about their sexual activity seven days after the procedure. Men without complications will be followed six months after surgery with behavioral risk assessment, HIV testing and counseling. Men may come to the study sites for unscheduled visits at any time during the 6 month follow-up. All men will be counseled at enrollment and all follow-up visits to reduce their risk for HIV infection by consistent condom use and, where applicable, reductions in numbers of sex partners. Counselling on condom use and sexual risk reduction will be provided by trained, experienced, native-speaking counsellors.

Study length and estimated sample size:

MC services will be offered at the selected two sites for 1 year. The estimated cost per circumcision is $40. We expect to recruit five clients per week at each site for a total convenience sample of 500 subjects in one year (250 per site). During the second year of the project, we will continue to assess behavioral risk factors for the first six months and work on analyzing the data during the last semester of the project.

<u>Specific aims #4 and #5:</u>

Circumcision Procedure

Prior to surgery, the clinician will obtain a complete medical history and perform a physical exam (form 3).

A brief description of the procedure follows. The patient lies on his back in a comfortable position. The pubic area is shaved clean with a disposable razor and the hair is picked up with a dressing tape. The surgeon scrubs up and puts on 2 pairs of sterile gloves. The client's skin is prepared with Betadine solution, making sure that the inner surface of the prepuce and the glans are clean and the skin is dry. The outer pair of gloves is then removed. The client is draped with two drapes and a center "O" towel, and then, using 2% lidocaine without epinephrine, a dorsal nerve block and a field block

with special attention to the ventral nerve, is performed. Normally, no more than 10ml of 2% lidocaine will be used. The anesthetic effect of the nerve block is checked, and revised if necessary. The prepuce is held with two mosquito forceps, one on each lateral aspect, then a curved mark is made with sterile disposable marking pens, outlining the planned surgical cut. The mark is made one cm proximal to the coronal sulcus all round, and parallel to the coronal sulcus. The prepuce is clamped along the mark with a Kocher clamp while retracting the glans, ensuring that the glans itself is not clamped. The prepuce is excised using a surgical blade along the mark. Bleeders are identified, clamped and tied. They are sutured, and if necessary ligated, using 3/0 plain catgut. After ligating all the bleeders, the area is irrigated with normal saline and then inspected for more bleeders, which are identified and tied. Using 3/0 chromic catgut on a taper 4/8 circle needle, a U-shaped horizontal mattress stitch is made on the ventral side of the penis (frenulum) to join the skin at the “V” shaped cut, which is then tied and tagged with a mosquito forceps. Using vertical mattress stitches, four quarters are tagged, and a vertical mattress stitch added, after which a simple stitch is put at the center of every two mattress stitches (a total of at least 16 stitches). The area is irrigated with normal saline and other simple stitches added as required. The wound is dressed with Sofratulle, then with a regular dressing bandage and a strapping. The client is advised to rest for 30 minutes, and if stable, discharged home on mild analgesics. Upon completion of the procedure the surgeon completes a procedure form (form 4). The excised foreskin tissue is taken to the laboratory with no identifiers, discarded with other laboratory waste, and incinerated with other biological waste within three days.

Participants will be given verbal and written instructions on postoperative wound care and counseled to refrain from sexual activity for at least six weeks after the procedure. They will also be encouraged to come to the clinic or contact a clinician at any time with medical problems.

Follow- up study visits

Participants will be offered the equivalent of US$ 5.00 for each scheduled study visit to cover travel expenses.

Day 7 Post Circumcision Visit (form #5)

Circumcised men will be asked to return to the clinic 7 days after the procedure to check the wound, to see if it is healing properly, to check for complications, and to ask about their sexual activity, their level of pain, resumption of work or other activities, and their satisfaction with the procedure and care at the clinic. As with all visits, any adverse events will be recorded. Blood and urine will be collected for STD testing at this visit.

Six month post operative visit (form #6)

During this visit a brief exam to confirmed proper wound healing will be performed. A behavioral and satisfaction questionnaire will be administered.

A semi-structured qualitative interview will be conducted to explore in-depth the lived experience of male circumcision. Interviews will be conducted by a trained qualitative researcher using a semi-structured guide in a private location at the clinic. Participants will be asked about their satisfaction with the procedure, perceptions about post-operative sexual performance, partner satisfaction and sexual risk behaviors, among other topics (Appendix B). Probing techniques will be used to elicit in-depth and detailed accounts of personal experiences and perceptions following circumcision and to limit socially desirable responses. Field notes will be used to provide additional insight into the context of the interview and any important non-verbal communication.

Qualitative data analysis will be iterative, starting with the completion of the first interviews and continuing throughout the data collection process. All transcripts will be analyzed in Spanish. Transcripts will be read multiple times by the study team to identify key themes and stories. A list of analytical codes will be developed based on the themes that emerge from the interview transcripts and the transcripts will be coded using the Atlas.ti software. The outputs of the coding exercises will be used to prepare matrices for the analysis of patterns across the study population and for comparisons between sub-groups (for example, older men vs younger men, married vs single). Memo writing will be integrated throughout the process to facilitate the interpretation of the data and to provide an audit trail of the analysis that can be used to document how the data were interpreted. Socio-demographic data will be summarized using SPSS to provide a descriptive sample of the study population.

Researchers at the University of North Carolina will be involved in this qualitative data collection and analysis. All data obtained and shared among performance sites will be de-identified. The names of the participants will not be recorded to protect their confidentiality.

Unscheduled Follow-up Visits

Ad hoc clinic visits will be encouraged to check and treat for complications of the procedure. At such visits, medical examinations and laboratory testing will be conducted as indicated, and appropriate treatment provided free of charge. Medical referral will be made if required.

<u>Outcomes</u>

The primary objectives of the proposed study are to assess the uptake, safety (adverse event rate) and patient satisfaction, train providers and build capacity on VMMC in the Dominican Republic. In addition, we will assess risk behaviors before and after VMMC and build key partnerships with the MoH of the DR and other stakeholders, which will

help develop a proposal to the NIMH to conduct operational research in the context of larger scale provision of MC services.

The study will assess the number of patients who undergo the procedure and the degree of post-operative satisfaction.

We will also record immediate complications of the circumcision procedure, which include: 1) pain, 2) excessive bleeding, 3) anesthetic-related complications, 4) excessive skin removal and 5) damage to the penis. Early post-surgical complications will include the occurrence of any of the following within one month of the procedure: 1) pain, 2) excessive bleeding, 3) excessive skin removed, 4) insufficient skin removed, 5) swelling or hematoma, 6) damage to the penis, 7) infection, 8) delayed wound healing, 9) problem with the appearance of the penis, and 10) problems with voiding.

The following complications will be evaluated at six months after the procedure: 1) infection, 2) delayed wound healing, 3) problem with the appearance of the penis, 4) excessive skin removed, 5) insufficient skin removed, 6) torsion of the penis, 7) erectile function problems, and 8) psycho-behavioral problems. In addition to the 8-day and six month post-surgery visits, men will be encouraged to come to the clinic for an unscheduled visit at any time they have excessive pain, they have bleeding, they detect an infection, or they desire treatment for any reason. Complications will be recorded at those unscheduled visits as well. See the sections on Adverse Events and Reporting Adverse Events below for more detail.

Sexual behavior will be another important outcome, particularly as there is concern that men who are circumcised may mistakenly feel that they are protected from HIV and may adopt more risky sexual behavior. On the other hand, sexual activity among circumcised men is likely to be much less for a period of time following the circumcision, which will decrease risk during that period. Self-reported sexual behavior (e.g. number of sex partners, lifetime, last 12 months and last 6 months; sex for gifts or money; condom use) will be assessed at baseline and at 6 months with a standard set of questions. We will also ask participants directly about their perceptions of change in risk for HIV acquisition.

Emergency Procedures

The major potential acute emergency resulting from the circumcision procedure is excessive bleeding. Standard surgical procedures will be used to stop bleeding during and following the procedure, including ligating bleeding vessels and the use of direct pressure. If bleeding cannot be controlled in this manner, then moderate pressure dressings will be applied to arrest bleeding. If this is not sufficient, high pressure dressings will be applied, if necessary in conjunction with a Foley catheter. Surgical re-exploration may also be indicated.

If bleeding continues despite these measures, an intravenous line will be inserted, a normal saline drip infused, and oxygen administered. The participant will be carried on a stretcher to an ambulance and transported to the Luis Eduardo Aybar Hospital in Santo Domingo or the Hospital Provincial Francisco Gonzalvo in La Romana for emergency attention. Both hospitals are very close to the respective study clinics. Oxygen and

equipment for the intravenous line are on hand in the operating room at the study clinic at all times. Arrangements have been made with the hospitals to receive the patients.

The other major potential complication of the procedure is an anaphylactic reaction to the local anesthetic. If this occurs, then supportive medical attention will be given, and subcutaneous epinephrine administered if indicated. If necessary, an intravenous line will be applied, oxygen administered, and the patient transferred to the referral hospital, as described above.

<u>Adverse Events</u>

The presence or absence of adverse events will be recorded at every visit. So as not to miss any event that could be considered adverse, we will apply a broad definition adapted from the definition and recorded in the ICH Guideline for Good Clinical Practice:

> *An adverse event is any untoward medical occurrence in a participant and that does not necessarily have a causal relationship with the treatment. An adverse event can therefore be any unfavorable and unintended sign (including an abnormal laboratory finding), symptom, or disease temporarily associated with the beginning of the treatment (i.e., following randomization).*

Adverse events will be characterized as mild, moderate, severe or death, and not related, possibly related, probably related or definitely related to the circumcision procedure.

The following table lists adverse events that are related to the intervention (circumcision procedure), according to the time that they will be ascertained, and provides definitions of severity. If death occurs as a consequence of an adverse event, this will be noted and recorded as well.

**Adverse Event Type**	**Description of Adverse Event Type**	**Severity**
**A. During Surgery**		
Pain	3 or 4 on pain scale	Mild
	5 or 6 on pain scale	Moderate
	7 on pain scale	Severe
Excessive bleeding	More bleeding than usual, but easily controlled	Mild
	Bleeding that requires pressure dressing to control	Moderate
	Blood transfusion or transfer to another facility for management required	Severe
Anesthetic-related event	Palpitations, vaso-vagal reaction or emesis	Mild
	Reaction to anesthetic requiring medical treatment in study clinic but not transfer to another facility	Moderate
	Anaphylaxis or any reaction requiring transfer to another facility	Severe

Excessive skin removed	Adds time or material needs to the procedure, but does not result in any discernable adverse condition	Mild
	Skin is tight, but additional operative work not necessary	Moderate
	Requires re-operation or transfer to another facility to correct the problem	Severe
Damage to the penis	Mild bruising or abrasion, not requiring treatment	Mild
	Bruise or abrasion to the glans or shaft of the penis requiring pressure dressing or additional surgery to control	Moderate
	Portion or all of the glans or shaft of the penis severed	Severe
**B. Post-Surgical complications**		
Pain	3 or 4 on pain scale	Mild
	5 or 6 on pain scale	Moderate
	7 on pain scale	Severe
Excessive bleeding	Dressing soaked through with blood at a routine follow-up visit	Mild
	Bleeding that requires a special return to the clinic for medical attention	Moderate
	Bleeding that requires surgical re-exploration to control	Severe
Excessive skin removed	Client concerned, but there is no discernable abnormality	Mild
	Skin is tight, but additional operative work not necessary	Moderate
	Requires re-operation or transfer to another facility to correct the problem	Severe
Insufficient skin removed	Prepuce partially covers the glans only when extended	Mild
	Prepuce still partially covers the glans and re-operation is required to correct	Moderate
	Not applicable	Severe
Swelling/Hematoma	More swelling than usual, but no significant discomfort	Mild
	Significant tenderness and discomfort; no or only minor surgical re-exploration required	Moderate
	Surgical re-exploration required to correct	Severe
Damage to the penis	Mild bruising or abrasion, not requiring treatment	Mild
	Bruise or abrasion to the glans or shaft of the penis requiring pressure dressing or additional surgery to	Moderate

	control	
	Portion or all of the glans or shaft of the penis severed	Severe
Infection	Erythema more than one cm beyond incision line	Mild
	Purulent discharge from the wound	Moderate
	Cellulitis or wound necrosis	Severe
Wound dehiscence	Dehiscence of 2 or less suture points requiring resuturing without further complications	Mild
	Dehiscence of 3-5 suture points requiring resuturing	Moderate
	Dehiscence of more than 5 suture points requiring resuturing with complicated postoperative course	Severe
Delayed wound healing	Healing takes longer than usual, but no extra treatment necessary	Mild
	Additional non-operative treatment required	Moderate
	Requires re-operation to correct	Severe
Appearance	Client concerned, but no discernable abnormality	Mild
	Significant wound disruption or scarring, but does not require re-operation	Moderate
	Requires re-operation to correct	Severe
Problems with voiding	Transient complaint that resolves without treatment	Mild
	Requires a special return to the clinic, but no additional treatment required	Moderate
	Requires referral to another facility for management	Severe
**C. One Month or More Post-Surgery**		
Infection	Erythema more than one cm beyond incision line and purulence in 2 or less suture points	Mild
	Purulent discharge from the wound in more than 2 suture points	Moderate
	Cellulitis or wound necrosis	Severe
Delayed wound healing	Healing takes longer than usual, but no extra treatment necessary	Mild
	Additional non-operative treatment required	Moderate
	Requires re-operation to correct	Severe
Appearance	Client concerned, but no discernable abnormality	Mild
	Significant scarring or other cosmetic problem, but does not require re-operation	Moderate
	Requires re-operation to correct	Severe
Excessive skin removed	Client concerned, but there is no discernable abnormality	Mild
	Skin is tight, but additional operative work not necessary	Moderate
	Requires re-operation or transfer to another facility to	Severe

	correct the problem	
Insufficient skin removed	Prepuce partially covers the glans only when extended	Mild
	Prepuce still partially covers the glans and re-operation is required to correct	Moderate
	Not applicable	Severe
Torsion of penis	Torsion is observable, but does not cause pain or discomfort.	Mild
	Causes mild pain or discomfort, but additional operative work not necessary	Moderate
	Requires re-operation or transfer to another facility to correct the problem	Severe
Erectile dysfunction	Client reports occasional inability to have an erection	Mild
	Client reports frequent inability to have an erection	Moderate
	Client reports complete or near complete inability to have erections	Severe
Psycho-behavioral problems	Client reports mild dissatisfaction with the circumcision, but no significant psycho-behavioural consequences	Mild
	Client reports significant dissatisfaction with the circumcision, but no significant psycho-behavioural consequences	Moderate
	Significant depression or other psychological problems attributed by the client to the circumcision	Severe

Appropriate medical care for adverse events will be provided to all study participants, as well as surgical care if required. If referral for medical or surgical complications are required, they will be made to the referral hospitals.

As indicated above, adverse events that are not or may not be related to the circumcision procedure will also be recorded. These will generally be ascertained by the project medical or clinical officer during a scheduled or unscheduled visit, either by history or physical examination. Severity will be judged as follows:

Mild: Transient or minimal symptoms; did not appreciably affect activity.

Moderate: Notable symptoms requiring modification of activity, but not resulting in loss of work or cancellation of social activities.

Severe: Incapacitating symptoms, requiring bed rest and/or resulting in loss of work or cancellation of social activities, and/or significant intervention therapy, including hospitalization.

All severe adverse events will be considered serious adverse events (SAEs) for reporting purposes (see below). This is consistent with the following FDA definition of serious adverse events:

Death

Life-Threatening: if the patient was at substantial risk of dying at the time of the adverse event.

Hospitalization (initial or prolonged): if admission to the hospital or prolongation of a hospital stay results because of the adverse event.

Disability: if the adverse event resulted in a significant, persistent, or permanent change, impairment, damage or disruption in the patient's body function/structure, physical activities or quality of life.

Intervention required to prevent permanent impairment or damage: if it is suspected that the use of a medical product may result in a condition which required medical or surgical intervention to preclude permanent impairment or damage to a patient.

Any SAEs which occur and which had not been classified as severe according to the study classification will also be reported as SAEs, as described below.

As the intervention is a surgical procedure, it will usually be obvious whether an adverse event is related or unrelated to the procedure. The following definitions will be used:

Definitely related: A clear complication of surgery has occurred either during or after surgery.

Probably related: A complication has occurred during or after surgery which is most likely explained by the circumcision procedure, but could possibly be explained by other causes.

Possibly related: A complication has occurred during or after surgery which might be explained by the circumcision procedure, but could equally or more likely be explained by other causes.

Not related: An adverse event has occurred which is clearly explained by a cause other than the circumcision procedure.

Determinations of adverse events, their severity and their relationship to the circumcision procedure will be made by the on-site study co-investigator.

Reporting Adverse Events

As indicated above, the attending physician (co-investigator) will determine whether an adverse event is serious. The physician performing the MC or examining the patient during a follow up visit, will inform the on-site co-investigator, in person, of the occurrence of an adverse event or death. This communication will occur verbally and in

person since these individuals will be working within the same clinic, several feet from each other. All deaths and AEs categorized as serious will then be reported to the P.I. promptly via e-mail by the Study co-investigator within two days of the event. The study co-investigator will complete and adverse event form and will fax to the P.I. shortly after the occurrence. The P.I. will then report the AE to the UIC and Dominican IRB within 48 hours. The relationship of the AE will be specified as definitely, probably, possibly or not related to study procedures. The occurrence and type of AE, its severity, and its relationship to the circumcision procedure will be determined by the project local co-investigator in consultation with the surgeon and the principal investigator. A summary of how the severity and relationship was determined will be provided to the P.I. In case of any question, the P.I. will speak with the project staff by telephone for clarification.

AEs will be delineated by type, number of days from surgery (day of surgery being Day 0), level of severity (mild, moderate, severe, death) and relationship to the study procedures. AEs will also be listed by date and type to detect any patterns in timing of AE. For example, if a cluster of infections is detected, a review of infection control procedures may be warranted.

d.2. Data analysis

<u>Data management</u>

Each enrolled participant will be assigned a confidential code, which will be used in all study forms. Only the principal investigators and research assistants will have access to the "locator information form" containing personal information. This form, which links the subject's confidential code to his name, will be stored in separate locked filing cabinets at the local principal investigator's offices. From that point on, all study forms, surveys and surgical forms completed on the subjects will only contain a number and will not be traceable to a specific individual. As clarified on our previously amended review application (see Initial Review Application, version #4, 4/27/11, section XIV.C.6. page 24), the locator information forms, which contain the personal information on the subjects, will be kept for 1.5 years in case we need to contact one of the subjects for an unexpected occurrence or with a question. After the end of that period, these locator forms will be destroyed at the research offices at the respective study sites using a micro cut shredder.

Data will be coded and entered into computer datasets by trained personnel. Data extracted from questionnaires will be tabulated and analyzed using SAS statistical software version 9.1. and will be analyzed at the University of Illinois, College of Medicine, in Chicago by the principal investigator

<u>Data analysis</u>

A questionnaire asking specific demographic information will be administered. The following variables will be recorded: age, religion, place of residence (urban/rural), education level, occupation and marital status. Simple descriptive statistics will be

calculated and the effect of these variables on the willingness, uptake and rate of adverse events of VMMC will be estimated. In addition, we plan to estimate the effect of demographic characteristics on sexual behavior and disinhibition after the procedure.

Specific aim #1:

The knowledge and beliefs of the subject about MC will be assessed by specific sections of the behavioral questionnaire . The patient will select from a list of proven benefits of the procedure (i.e. HIV/STI risk reduction) and risks (i.e. surgical complications). The responses will be analyzed to determine the number of patients who correctly identify the benefits of the procedure and the most common adverse events. We will be able to assess their willingness to be circumcised as a function of their level of knowledge and understanding of the risk and benefits.

Specific aim #2:

The length of the procedure, technical aspects of the procedure and perioperative complications will be recorded. Analysis of this data will allow estimation of the types of adverse events (AEs), operative complication rate and median length of surgery.

Specific aims #3 and #4:

Patient satisfaction with VMMC will be assessed at every postoperative visit. Forms 6 and 7 ask clients about their satisfaction or dissatisfaction with the procedure and their partners acceptability of their VMMC. Subjects will also be asked if they have recommended VMMC to other men. If so, we will record and estimate the most common reason for recommending the procedure to their peers. Other outcomes to be recorded and analyzed include: number of men requesting VMMC by age; number circumcised by age and marital status; proportion circumcised who are HIV tested and counseled; time and personnel involved in each surgery; proportion of circumcisions with moderate and severe AEs by day of ascertainment, and proportion of men who resume sex before 42 days post-surgery.

Specific aim #5:

Specific questions on sexual practices, time of resumption of sexual activity, sexual pleasure after VMMC and partner’s sexual satisfaction will be asked at follow up visits. This will allow the estimation of the proportion of men who resume sex earlier than recommended and the effects of VMMC on sexual pleasure. Behavioral disinhibition will be estimated by analyzing the subject’s self perceived level of protection against HIV/STI and their risk taking behavior (i.e. low rates of condom use).

d.3. Serum sample management

A sample of the patient's serum will be aliquoted and cryopreserved at -65 C to -90 C following published Standard Operation Procedures at the Instituto Dermatologico y Cirugia de Piel (IDCP) in Santo Domingo, Dominican Republic. The sample will only be labeled with the study ID. When all samples of the study have been collected, they will be transported in a single shipment, preserving the cold chain, to the University of Illinois Division of Infectious Diseases for cryopreservation. No personal information will be transported with the samples. For transportation, we will use an authorized courier experienced with transporting samples internationally in and out of our UIC laboratories. The personal information of the subject (locator information form) will remain in the Dominican Republic. As long as we hold personal identifiable information on the subjects, we will contact them at the phone number provided in the locator form to inform them of any results of future testing. We will provide them with a paper copy of their test results free of charge. Since we plan to destroy all personal information before we close the study, results of testing performed after this destruction of personal information takes place will not be provided to the subjects. After destruction of all identifiable information and prior to the study closing, the samples will be transferred and stored deidentified in a biobank. While we do not currently have a biobank established, we are aware that a biobank needs to be established prior to the completion of the research. We will establish the biobank using the necessary procedures and guidelines. Samples will continue to be stored deidentified in this biobank until all available sera has been consumed and there is no sera left to perform further testing**.** These samples will not be used for genetic testing.

d.4.Institutional Review Boards for this study

The study will be presented for approval to the Institutional Review Boards of the University of Illinois at Chicago, The IDCP and the Consejo Nacional de Bioetica (Bioethics National Council) of the Dominican Republic.

e) Resources and collaborators:

The pilot study will be conducted in 2 clinics, one urban and one rural, serving high risk populations in the Dominican Republic. One of the sites will be the STI clinic at the IDCP, located in the capital city of Santo Domingo. This clinic is the largest provider of STI diagnostic and treatment services in the country and serves a high risk urban population. In 2009, 19,150 patients (7,037 males and 12,133) sought care for a STI at the clinic[24]. The IDCP has 8 satellite clinics around the country, which provide care to low income patients with STI. The main STI clinic in Santo Domingo is an HIV vaccine research site of the HIV Vaccine Trials Network (HVTN). The IDCP has modern, fully equipped and functioning surgical facilities used mostly for dermatologic surgeries. Space and basic surgical instruments are available at the center. Data forms will be kept in a locked filing cabinet at the research offices of the IDCP. Database will be maintained by the research assistance using a laptop computer that belongs to the

principal investigator. This site will have two local co-investigators who currently work at the IDCP- Dr. Claudio Volquez, is the medial director of the STI clinic. He is a dermato-venerologist with more than 20 years of experience in the field. He is a co-investigator for the HIV vaccine trial for which he has received NIH support and training in the past. Yeycy Donastorg, MPH is the principal investigator for the Dominican site of the HVTN. She has ample experience conducting NIH funded studies and has the infrastructure, knowledge and skills for the local supervision of the research work.

The Clínica de Familia, serving the city of La Romana and the bateyes of Central Romana Sugar Cane Corporation. The site was chosen for several important reasons including our previous findings, which suggest a high degree of acceptability among inhabitants; the mixed population of Dominicans and Haitian migrant workers, which will allows a comparison between nationalities; and a high prevalence of HIV (3.2%) secondary to high risk heterosexual practices in men due to frequent contact with CSW. The site will be monitored by the director of the clinic, Dr. Leonel Lerebours, and a research assistant who will be hired part-time for this project. Space and essential surgical equipment are available at the clinic. Our team will supplement any surgical instrument that is lacking.

Dr. Max Brito, the principal investigator in this proposal, will be responsible for direct oversight of the project, data analysis and writing of the manuscripts. Dr. Robert C. Bailey, will be the senior co-investigator in the project. He will provide mentorship, guidance, will review the data and will contribute to the writing of the manuscripts. Several MoH officials of the DR are collaborating with our projects. Dr. Luis E. Feliz Baez is the Director of the Office for the Control of Sexually Transmitted Diseases and AIDS in Santo Domingo and Dr. Jaime Rodriguez is the Provincial Director for the Ministry of Health in Region V of health.

References

1. Auvert B, Taljaard D, Lagarde E et al. Randomized, controlled intervention trial of Male Circumcision for reduction of HIV infection risk: the ANRS 1265 trial. PloS Med 2005; 2: 1112-22.

2. Gray RH, Kigozi G, Serwadda D et al. Male circumcision for HIV prevention in men in Rakai, Uganda: a randomised trial. Lancet 2007; 369: 657-66.

3. Bailey RC, Moses S, Parker CB et al. Male circumcision for HIV prevention in young men in Kisumu, Kenya: a randomised controlled trial. Lancet 2007; 369: 643-56.

4. Tobian, A. A., Serwadda, D., Quinn, T. C., Kigozi, G., Gravitt, P. E., & Laeyendecker, O., et al. . Male circumcision for the prevention of HSV-2 and HPV infections and syphilis. N Engl J Med 2009. 360; 1298–1309.

5. Castellsague, X., Peeling, R. W., Franceschi, S., de Sanjose, S., Smith, J. S., & Albero, G., et al. . Chlamydia trachomatis infection in female partners of circumcised and uncircumcised adult men. Am J Epidemiol 2005. 162(9), 907–916.

6. Gray, R. H., Kigozi, G., Serwadda, D., Makumbi, F., Nalugoda, F., & Watya, S., et al. The effects of male circumcision on female partners' genital tract symptoms and vaginal infections in a randomized trial in Rakai, Uganda. American Journal of Obstetrics & Gynecology 2009; e1–e42.

7. Drain PK, Halperin DT, Hughes JP, Klausner JD, Bailey RC. Male circumcision , religion, and infectious diseases: an ecologic analysis of 118 developing countries. BMC infectious diseases 2006; 6: 172-82.

8. CESDEM. Encuesta Demográfica y de Salud, República Dominicana. 2007. (accessed January 2, 2010 at (http://www.cesdem.com/html/encuesta_demografica_y_de_salud_2007.pdf)

9. Joint United Nations Programme on HIV /AIDS. 2009 Report on the global AIDS epidemic. (Accessed February 2, 2010 , at http://www.unaids.org/en/KnowledgeCentre/HIVData/GlobalReport/

10. CESDEM. Encuesta Sociodemográfica y sobre VIH/SIDA en los Bateyes Estatales de la República Dominicana. 2007. (accessed January 2, 2009 at http://www.cesdem.com/html/encuesta_sociodemografica_y_sobre_vih_sida_en_los_bateyes_estatales_de_la_republica_dominicana.pdf)

11. Secretaria de Estado de Salud Publica. Encuesta de seroprevalencia de la infección VIH basadas en puestos centinela. 2006.

12. UNAIDS. Male circumcision: global trends and determinants of prevalence, safety and acceptability. (accessed February 23, 2010 at (http://whqlibdoc.who.int/publications/2007/9789241596169_eng.pdf)

13. Weiss HA, Quigley MA, Hayes RJ. Male circumcision and risk of HIV infection in Sub-Saharan Africa: a systematic review and meta-analysis. AIDS 2000; 14:2361-70.

14. Kahn JO, Walker BD. Acute human immunodeficiency virus type 1 infection. N Engl J Med 1998; 339; 33-39.

15. McCoombe SG, Short RV. Potential HIV-1 target cells in the human penis. AIDS 2006; 20: 1491-95.

16. Gray RH, Serwadda D, Tobian AAR, Chen MZ, Makumbi F, et al. Effects of Genital Ulcer Disease and Herpes Simplex Virus Type 2 on the Efficacy of Male Circumcision for HIV Prevention: Analyses from the Rakai Trials. PLoS Med 2009. 6: e1000187. doi:10.1371/journal.pmed.1000187

17. ONE. VIII Censo nacional de poblacion y vivienda 2002. (accessed February 23, 2010 at(http://onedatabase.indotel.net.do/cgibin/RpWebEngine.exe/PortalAction?&MODE=MAIN&BASE=CPV2002&MAIN=WebServerMain.inl)

18. COPRESIDA. Primera encuesta de vigilancia de comportamiento con vinculacion serologica en poblaciones vulnerables 2008. 1: 39-88.

19. Kbaabetswe P, Lockman S, Mogwe S, Mandevu R, Thior I et al. Male Circumcision: an acceptable strategy for HIV prevention in Botswana. Sex Trans Infect 2003; 79: 214-19.

20. H.V.Tieu, et al. Acceptability of male circumcision for prevention of HIV among high-risk heterosexual men in Thailand : AIDS 2008 - XVII International AIDS Conference: Abstract no. MOPE0547.

21. Brito MO, Caso LM, Balbuena H, Bailey RC (2009) Acceptability of Male Circumcision for the Prevention of HIV/AIDS in the Dominican Republic. PLoS ONE 4(11): e7687. doi:10.1371/journal.pone.0007687

22. Brito M, Luna M, Bailey RC. Feasibility of introducing male circumcision (MC) in the Dominican Republic : AIDS 2008 - XVII International AIDS Conference: Abstract no. MOPE0548.

23. WHO. Manual for MC under local anesthesia 2009. (accessed February 23, 2010 at(http://www.who.int/hiv/pub/malecircumcision/local_anaesthesia/en/index.html)

24. Departamento de Estadistica. Instituto Dermatologico. Resumen annual de pacientes nuevos y subsecuentes, 2009.

25. WHO. Operational guidance for scaling up MC services for HIV prevention. (accessed February 23, 2010 at(http://www.who.int/hiv/pub/malecircumcision/op_guidance/en/index.html)

26. WHO. Male circumcision and HIV prevention: operations research implications. (accessed February 23, 2010 at(http://www.who.int/hiv/pub/malecircumcision/meetingreport_jun07/en/index.html)

Timeline

Activity	Months																				
	1	2	3	4	5	6	7	8	9	10	11	12	13	14	15	16	17	18	19	20	21	22-24
Development of educational material	X																					
Training of providers		X	X																			
Recruitment of participants and Implementation			X	X	X	X	X	X	X	X	X	X	X	X	X							
Acceptability and uptake			X	X	X	X	X	X	X	X	X	X	X	X	X							
Safety and adverse AEs			X	X	X	X	X	X	X	X	X	X	X	X	X	X	X	X	X	X	X	X
Sexual behaviors			X	X	X	X	X	X	X	X	X	X	X	X	X	X	X	X	X	X	X	X
Analysis of data																X	X	X				
Preparation of manuscripts															X	X	X	X	X	X	X	X
Prepare R-O1 application																				X	X	X
